# Cell surface GRP78 regulates TGFβ1-mediated profibrotic responses *via* TSP1 in diabetic kidney disease

**DOI:** 10.3389/fphar.2023.1098321

**Published:** 2023-02-24

**Authors:** Jackie Trink, Usman Ahmed, Kian O’Neil, Renzhong Li, Bo Gao, Joan C. Krepinsky

**Affiliations:** Division of Nephrology, McMaster University, Hamilton, ON, Canada

**Keywords:** Thrombospondin 1, cell surface GRP78, fibrosis, mesangial cell, diabetic kidney disease, TGFβ1

## Abstract

**Introduction:** Diabetic kidney disease (DKD) is the leading cause of kidney failure in North America, characterized by glomerular accumulation of extracellular matrix (ECM) proteins. High glucose (HG) induction of glomerular mesangial cell (MC) profibrotic responses plays a central role in its pathogenesis. We previously showed that the endoplasmic reticulum resident GRP78 translocates to the cell surface in response to HG, where it mediates Akt activation and downstream profibrotic responses in MC. Transforming growth factor β1 (TGFβ1) is recognized as a central mediator of HG-induced profibrotic responses, but whether its activation is regulated by cell surface GRP78 (csGRP78) is unknown. TGFβ1 is stored in the ECM in a latent form, requiring release for biological activity. The matrix glycoprotein thrombospondin 1 (TSP1), known to be increased in DKD and by HG in MC, is an important factor in TGFβ1 activation. Here we determined whether csGRP78 regulates TSP1 expression and thereby TGFβ1 activation by HG.

**Methods:** Primary mouse MC were used. TSP1 and TGFβ1 were assessed using standard molecular biology techniques. Inhibitors of csGRP78 were: 1) vaspin, 2) the C-terminal targeting antibody C38, 3) siRNA downregulation of its transport co-chaperone MTJ-1 to prevent GRP78 translocation to the cell surface, and 4) prevention of csGRP78 activation by its ligand, active α2-macroglobulin (α2M*), with the neutralizing antibody Fα2M or an inhibitory peptide.

**Results:** TSP1 transcript and promoter activity were increased by HG, as were cellular and ECM TSP1, and these required PI3K/Akt activity. Inhibition of csGRP78 prevented HG-induced TSP1 upregulation and deposition into the ECM. The HG-induced increase in active TGFβ1 in the medium was also inhibited, which was associated with reduced intracellular Smad3 activation and signaling. Overexpression of csGRP78 increased TSP-1, and this was further augmented in HG.

**Discussion:** These data support an important role for csGRP78 in regulating HG-induced TSP1 transcriptional induction via PI3K/Akt signaling. Functionally, this enables TGFβ1 activation in response to HG, with consequent increase in ECM proteins. Means of inhibiting csGRP78 signaling represent a novel approach to preventing fibrosis in DKD.

## 1 Introduction

As the largest cause of kidney failure worldwide, diabetic kidney disease (DKD) places a significant burden on our healthcare system. Current therapies include stabilizing blood glucose and blood pressure, the use of inhibitors of the renin-angiotensin system and SGLT2 inhibitors in type 2 diabetics. Further studies are currently assessing efficacy of newer therapies such as inhibitors of DPP-4 and Rho-kinase, as well as the immunomodulatory peptide AcSDKP and activation of the sirtuin SIRT3 (Omata et al., 2006; Kolavennu et al., 2008; Castoldi et al., 2013; Zuo et al., 2013; Peng et al., 2016; Lin et al., 2018; Xu et al., 2018; Locatelli et al., 2020). However, the current standard of care for DKD can only slow disease progression, with many patients developing end-stage kidney disease and requiring costly therapies including dialysis or kidney transplant (Johnson and Spurney, 2015; Tuttle et al., 2020). Thus, identifying novel therapeutic interventions that can prevent disease progression is crucial. The primary hallmark of DKD manifestation begins with structural changes to the glomerulus, the filtering unit of the kidney. These pathological changes include glomerular basement membrane thickening and the overproduction of extracellular matrix proteins (ECM) by glomerular mesangial cells (MC) which leads to glomerulosclerosis and ultimately loss of filtration ability (Reidy et al., 2014; Alicic et al., 2017; Sagoo and Gnudi, 2020). Thus, MC play a critical role in the pathogenesis of DKD and are an important therapeutic target for attenuating fibrosis.

The profibrotic cytokine transforming growth factor β1 (TGFβ1) is well characterized for its role in promoting ECM production by MC as well as other kidney cell types including endothelial cells, fibroblasts and podocytes in response to high glucose (HG) through Smad-dependent and -independent signaling pathways (Verrecchia et al., 2001; Li et al., 2003; Edeling et al., 2016). Some of these latter pathways include Wnt, hedge-hog, SIRT3, FGFR1, glucocorticoid receptor-mediated signaling and PI3k/Akt. Together, these and others contribute to cellular transformation to a profibrotic phenotype (such as macrophage-to-myofibroblast transition (MMT), epithelial mesenchymal transition (EMT) and endothelial-to-mesenchymal transition (EndMT) (Kato et al., 2006; Lebleu et al., 2013; Nikolic-Paterson et al., 2014; Cho et al., 2018). TGFβ1 is secreted in an inactive form, with the mature cytokine non-covalently attached to its N-terminal propeptide, the latency-associated peptide (LAP). As LAP binding blocks TGFβ1 receptor binding, extracellular dissociation from LAP is required to reveal the receptor recognition site and thus cytokine activation (Munger et al., 1997; Koli et al., 2001). Integrin activation may dissociate LAP either through traction and mechanical release or through protease activation and LAP cleavage (Worthington et al., 2011). However, activation of TGFβ1 by HG in MC was shown to be dependent on the extracellular glycoprotein thrombospondin 1 (TSP1), which enables the non-proteolytic release of active TGFβ1 (Crawford et al., 1998; Yevdokimova et al., 2001). This interaction with LAP is specific to TSP1, as TSP2 lacks the peptide sequence required to non-proteolytically release and activate TGFβ1 (Hugo, 2003).

TSP1 interacts with both the mature portion of TGFβ1 and with LAP through distinct sites. Binding to the mature domain orients TSP1 to enable additional binding to LAP. This binding disrupts the intramolecular LAP-mature domain interaction to expose the receptor binding sequence of TGFβ1, enabling receptor binding and signaling (Murphy-Ullrich and Suto, 2018). Upregulation and increased deposition of TSP1 into the ECM as well as its role in TGFβ1 activation has been reported in HG in MC as well as *in vivo* in DKD mouse models and in human DKD (Poczatek et al., 2000; Wahab et al., 2005; Hohenstein et al., 2008; Lu et al., 2011). Furthermore, TSP1 knockout mice were protected from the development of DKD (Daniel et al., 2007), and the use of the peptide LSKL which blocks the interaction of TSP1 with LAP, protected type 1 diabetic Akita mice from DKD (Lu et al., 2011). Reduced active TGFβ1 and downstream signaling were seen in both studies. Thus, TSP1 is critical for the activation and profibrotic signaling of TGFβ1, but how TSP1 is upregulated in DKD has not yet been elucidated.

Previously, our lab has shown that the endogenous endoplasmic reticulum resident GRP78 translocates to the cell surface of MC in response to HG. Here, it acts as a receptor for the activated form of the protease inhibitor alpha 2 macroglobulin, the binding of which enables HG-induced downstream profibrotic signaling (Van Krieken et al., 2019; Trink et al., 2021; Trink et al., 2022). PI3K/Akt is a key mediator of cell surface (cs)GRP78 signaling, a pathway known to facilitate TGFβ1 synthesis as well as ECM production in response to HG (Wu et al., 2009; Van Krieken et al., 2019). We have also shown that csGRP78 inhibition attenuates the HG-induced synthesis of TGFβ1 (Trink et al., 2022). However, whether csGRP78 also contributes to TGFβ1 activation in HG through regulation of its non-proteolytic activator TSP1 has not yet been determined and is addressed in these studies.

## 2 Materials and methods

### 2.1 Cell culture

Primary MC from C57Bl/6 mice were isolated for culture using Dynabeads. They were cultured in DMEM (1,000 mg/L or 5.6 mM glucose) supplemented with 20% FBS, 100 µg/mL streptomycin, and 100 µg/mL penicillin at 37°C in 95% O_2_, 5% CO_2_. Cells were serum-deprived in 0.5% FBS for 24 h prior to treatment with HG (30 mM) with or without inhibitors of csGRP78 signaling: the C-terminus targeting GRP78 antibody C38 (2 µg/mL) (Munro and Pelham, 1987; De Ridder et al., 2012; Trink et al., 2022) or vaspin (100 ng/mL) (Nakatsuka et al., 2012; Nakatsuka et al., 2013; Abdolahi et al., 2022), or the following PI3K/Akt inhibitors: wortmannin (100 ng/mL), LY294002 (20 µM), and Akt Inhibitor VIII (10 µM).

### 2.2 Protein extraction and immunoblotting

MC protein extraction was previously described (Krepinsky et al., 2003). Protein expression was assessed using SDS-PAGE and immunoblotting. Antibodies used for Western blotting were: TSP1 (1:1,000, R&D Systems), pAkt Ser473 (1:1,000, Cell Signaling), total Akt (1:1,000, Cell Signaling), pSmad3 Ser423/425 (1:4,000, Novus), total Smad3 (1:1,000, Abcam), MTJ1 (1:1,000, Cedarlane), LAP-TGFβ1 (1:1,000, R&D Systems), GRP78 (1:1,000, BD Biosciences), α-tubulin (1:40,000, Sigma).

### 2.3 Luciferase and transfection

For transfection experiments, MC were plated at 50% confluency and transfected with either 1 µg of the mouse TSP1 luciferase reporter construct [mTSP1-luciferase, a gift from P. Bornstein, Plasmid #12409, Addgene (Michaud-Levesque and Richard, 2009)] with 0.05 µg pCMV β-galactosidase (β-Gal, Clonetech) using Effectene (Qiagen) or 100 nM of MTJ1 or control siRNA (Silencer Select, ThermoFisher) using Lipofectamine (Invitrogen). After 18 h, cells were serum-deprived and treated as above for protein collection for siRNA experiments. For luciferase harvest, 1× Reporter Lysis Buffer (Promega) was added to the plate which was then stored at −80°C overnight prior to cell lysis. Luciferase activity was measured on clarified lysate using the Luciferase Assay System (Promega) with a luminometer (Junior LB 9509, Berthold). Β-Gal activity was used to normalize transfection efficiency, measured using the β-Galactosidase Enzyme Assay System (Promega) with a SpectraMax Plus 384 Microplate Reader (Molecular Devices) set to read absorbance at 420 nm.

The pcDNA3.1 plasmid which contains GRP78 lacking its ER retention sequence KDEL was transfected by electroporation as previously described (Trink et al., 2022). This was used to overexpress GRP78 at the cell surface. The empty vector pcDNA 3.1 was used as a control.

### 2.4 RNA extraction and qtPCR

RNA was extracted from MC using Trizol (Invitrogen), and 0.5 µg of RNA was reverse transcribed using qScript Supermix Reagent (Quanta Biosciences). Expression of TSP1 mRNA relative to 18S was determined using the ΔΔCt method. Quantitative PCR was performed using Power SYBR Green PCR Master Mix on the Applied Biosystems Vii 7 Real-Time PCR System. The following primers were used: TSP1 forward 5′-TGGCCAGCGTTGCCA -3′ and reverse 5′- TCT​GCA​GCA​CCC​CCT​GAA-3′ and 18S forward 5′- GCC​GCT​AGA​GGT​GAA​ATT​CTT​G-3′ and reverse 5′- CAT​TCT​TGG​CAA​ATG​CTT​TCG-3’.

### 2.5 ELISA for active TGFβ1

To measure biologically active TGFβ1 in conditioned MC media, the TGFβ1 Quantikine ELISA kit (R&D Systems) was used, with omission of the activation step described in the protocol.

### 2.6 TGFβ1 bioassay with mink lung epithelial cells (MLECs)

MLEC stably transfected with the PAI-1 luciferase promoter construct, generously provided by Dr. T. Tsuda, were used. MC and MLEC were cocultured in MEM with 10% FBS, plated on a 12-well plate at 5,000 and 25,000 cells/well, respectively (1:5 ratio MLEC: MC). The following day, cells were serum deprived for 18 h followed by treatment with HG and various inhibitors. At collection, cells were lysed in 1× Reporter Lysis Buffer (Promega) and stored at −80°C overnight before analysis of PAI-1 luciferase activity as described above.

### 2.7 Extracellular matrix extraction

MC were lysed with 0.5% sodium deoxycholate [DOC; 0.5% DOC, 50 mM Tris pH 8.0, 150 mM NaCl, 1% Nonidet P-40; as used in (Klingberg et al., 2014)] three times to allow complete removal of cells while maintaining ECM adhesion to plates. Plates were then washed twice with cold DOC lysis buffer and three times with cold 1xPBS. Lysis buffer (PBS pH 7.4, 5 mM EDTA, 5 mM EGTA, 10 mM sodium pyrophosphate, 50 mM NaF, 1 mM NaVO3, 1% Triton) containing 1 mM DTT, 60 mM n-octyl glucopyranoside, and protease inhibitors was heated at 100°C for 2 min before being added to the plate. After thorough scraping, ECM lysate was transferred into an Eppendorf tube and boiled for an additional 10 min. Samples were assessed using SDS-PAGE and immunoblotting. An aliquot from the first DOC extraction of each sample was taken and run alongside the final ECM extraction to confirm the complete removal of cellular debris by probing for tubulin.

### 2.8 Surface protein co-immunoprecipitation from live cells

MC were washed three times with 1xPBS and then incubated in 1% BSA with 5 µg anti-TSP1 antibody at 4°C for 2 h on a shaker set to low speed. Cells were then washed and lysed by passing through a 25-gauge needle (Precision Glide Needle, B) 5 times. Lysates were clarified and normalized with an equal amount of Protein G beads (rProtein G agarose, Invitrogen) added to each sample with gentle rocking overnight at 4°C. Samples were then washed in lysis buffer and eluted from the beads by boiling for 5 min in PSB. Samples were assessed by SDS-PAGE and immunoblotting.

### 2.9 Statistical analysis

All points presented in graphs represent individual data points. A two-tailed *t*-test or one-way ANOVA was used to analyze differences between two or more groups, respectively. Tukey’s *post hoc* analysis was used to compare differences between two or more groups. ImageJ was used for the quantification of experiments and GraphPad Prism 6.0 was used for the analysis of data. Statistical significance was set to *p* < 0.05 and data are presented as mean ± SEM.

## 3 Results

### 3.1 PI3K/Akt signaling is required for HG-induced TSP1 regulation

**FIGURE 1:**
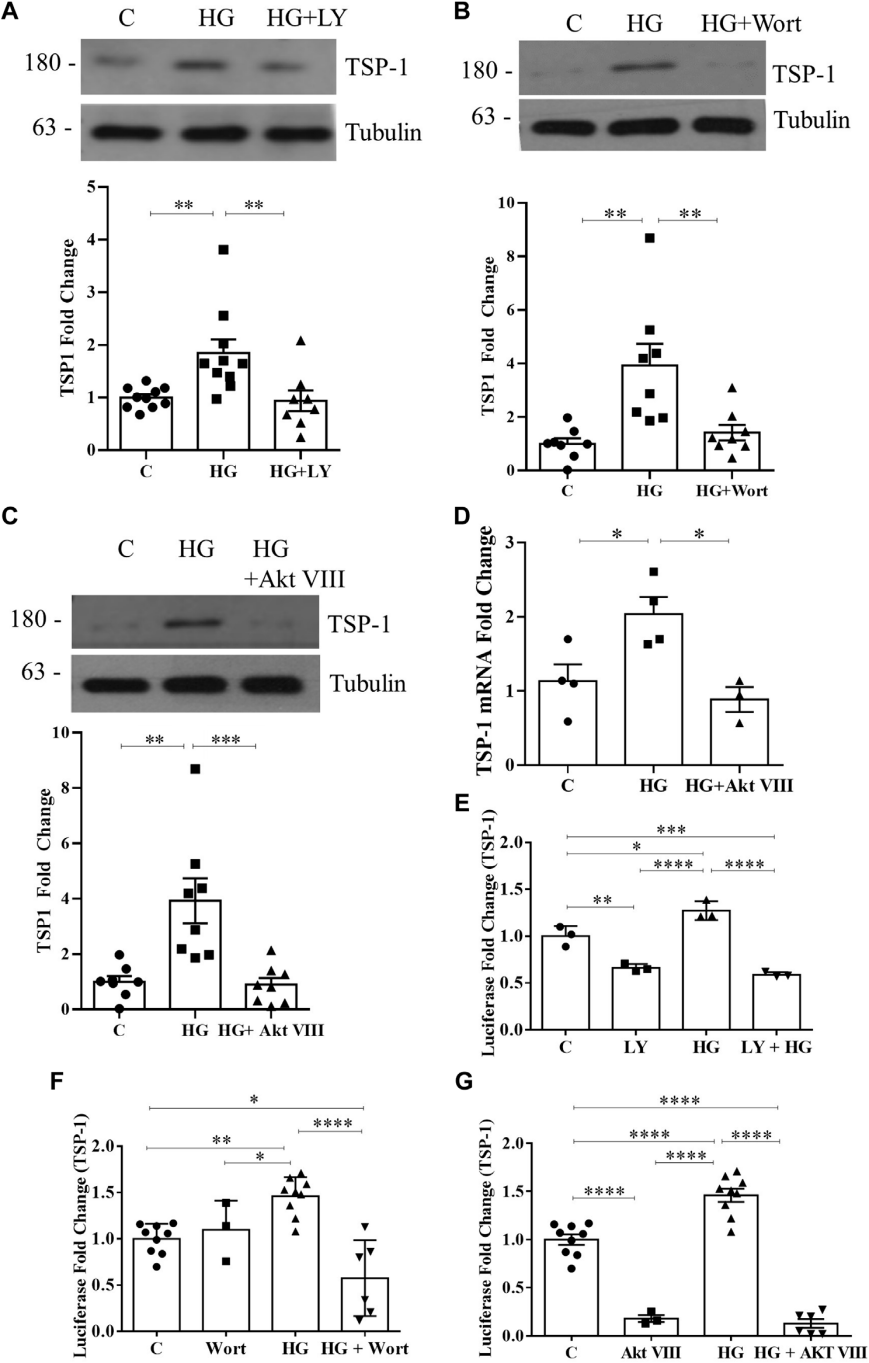
HG-induced TSP1 upregulation requires PI3K/Akt. HG (48 h)-induced TSP1 upregulation in MC was attenuated by the PI3K inhibitors **(A)** LY294002 and **(B)** wortmannin as well as **(C)** Akt inhibitor VIII (*n* = 10, **p < 0.01, ***p < 0.005). **(D)** TSP1 transcript upregulation by HG (24 h) was inhibited by Akt inhibitor VIII (*n* = 4, *p < 0.05). HG (48 h)-induced TSP1 promoter activity, assessed using a luciferase reporter construct, was also inhibited by **(E)** LY294002 (*n* = 3, *p < 0.05, **p < 0.01, ***p < 0.005, ****p < 0.0001), **(F)** wortmannin (*n* = 9, *p < 0.05, **p < 0.01, ****p < 0.001), or **(G)** Akt inhibitor VIII (*n* = 9, ****p < 0.001) in MC.

Previously we showed that HG-induced PI3K/Akt activation requires csGRP78 in MC (Van Krieken et al., 2019; Trink et al., 2021). Since PI3K/Akt signaling was shown to mediate TSP1 expression by complement in MC (Wang et al., 2006), we hypothesized that csGRP78 would promote TSP1 expression in HG through this pathway. To first test whether PI3K/Akt are required for HG-induced TSP1 upregulation, we used the following inhibitors: LY294002 and wortmannin to inhibit PI3K activity and Akt inhibitor VIII to inhibit Akt activity. We previously showed that the osmotic control mannitol has no effect on the cell surface translocation of GRP78 or the upregulation and activation of α2M in MC (Van Krieken et al., 2019; Trink et al., 2021). Further, mannitol does not induce TSP1 expression (Yevdokimova et al., 2001). Thus, mannitol was omitted from these experiments. We observed significantly increased TSP1 expression in response to HG, which was attenuated by all three inhibitors (Figures 1A–C). We next assessed TSP1 transcript regulation. Similar to its protein expression, HG-induced TSP1 transcript upregulation was attenuated by the Akt inhibitor (Figure 1D). Next, we confirmed that TSP1 promoter activity was also regulated by PI3K/Akt signaling. Both PI3K inhibitors and the Akt inhibitor suppressed TSP1 promoter activation by HG. Some suppression of basal activity was also seen with LY294002 and Akt VIII (Figures 1E–G). Taken together, PI3K/Akt is required for HG-induced TSP1 regulation.

### 3.2 PI3K/Akt inhibition attenuates HG-induced TGFβ1 activation and signaling

**FIGURE 2:**
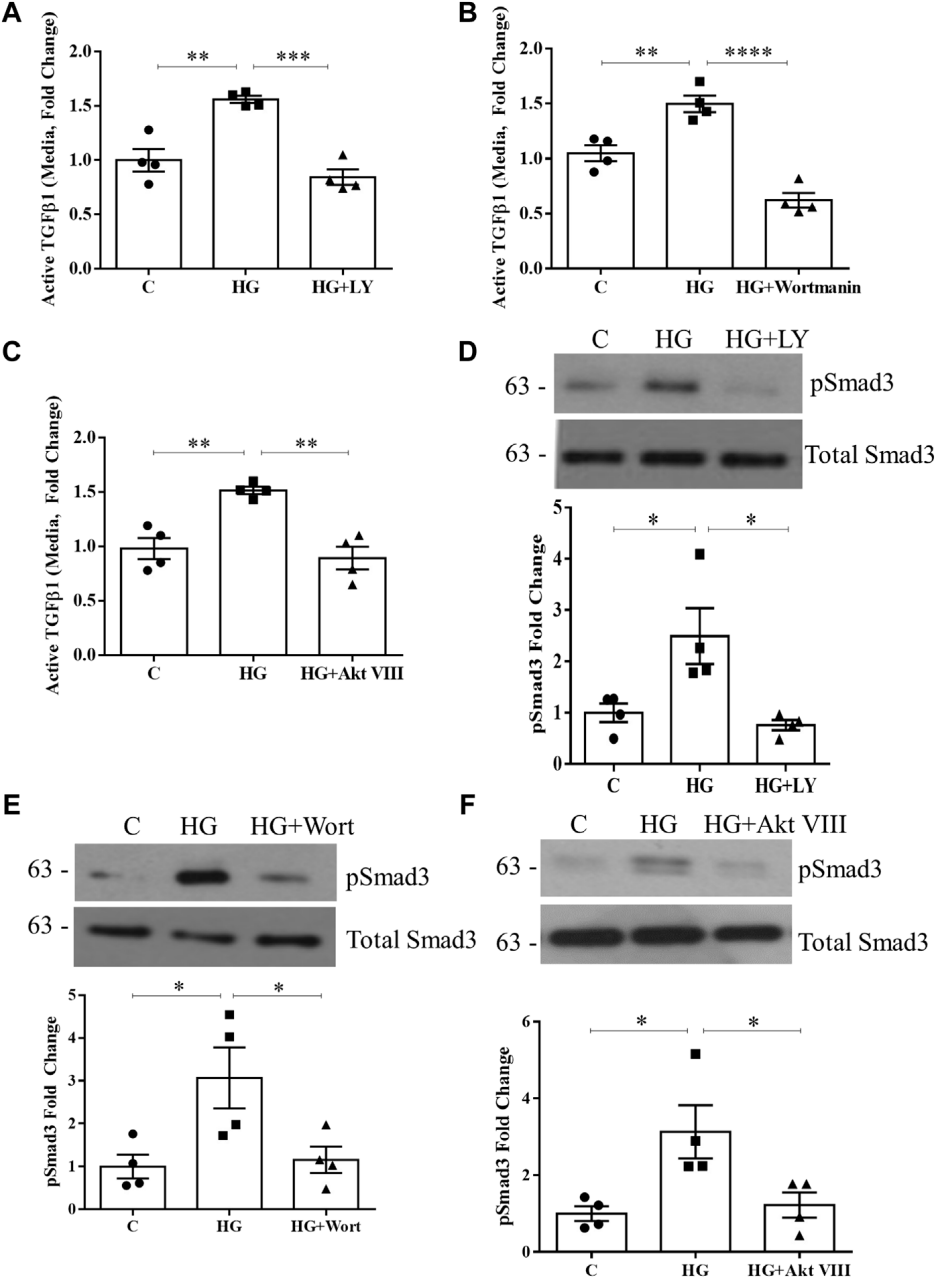
HG-induced TGFβ1 activation and signaling are blocked by PI3K/Akt inhibition. HG (48 h)-induced TGFβ1 activation, assessed by ELISA of medium without acid activation, was prevented by PI3K inhibition using either **(A)** LY294002 (*n* = 4, **p < 0.01, ***p < 0.005) or **(B)** wortmannin (*n* = 4, **p < 0.01, ****p < 0.0001), as well as Akt inhibition using **(C)** Akt inhibitor VIII (*n* = 4, **p < 0.01). Smad3 signaling downstream of TGFβ1 was assessed by its C-terminal phosphorylation at Ser473/475 (pSmad3). HG (48 h)-induced Smad3 phosphorylation was prevented by **(D)** LY294002, **(E)** wortmannin, and **(F)** Akt inhibitor VIII (*n* = 4, *p < 0.05).

Given that TSP1 is an important activator of TGFβ1 in response to HG (Yevdokimova et al., 2001), we next assessed whether PI3K/Akt inhibition would also abolish TGFβ1 activation and downstream signaling. In Figures 2A–C, the increase in active TGFβ1 in the medium seen with HG, as assessed by ELISA, was prevented by both PI3K and Akt inhibitors. Activation of the major mediator of TGFβ1 signaling Smad3 was then assessed by immunoblotting for its activated form, phosphorylated at its C-terminus Ser473/475. As anticipated, PI3K and Akt inhibition blocked HG-induced Smad3 activation (Figures 2D–F). Thus, PI3K/Akt are required for TSP1-mediated activation of TGFβ1 and its downstream signaling.

### 3.3 csGRP78 mediates HG-induced TSP1 expression through Akt activation

**FIGURE 3:**
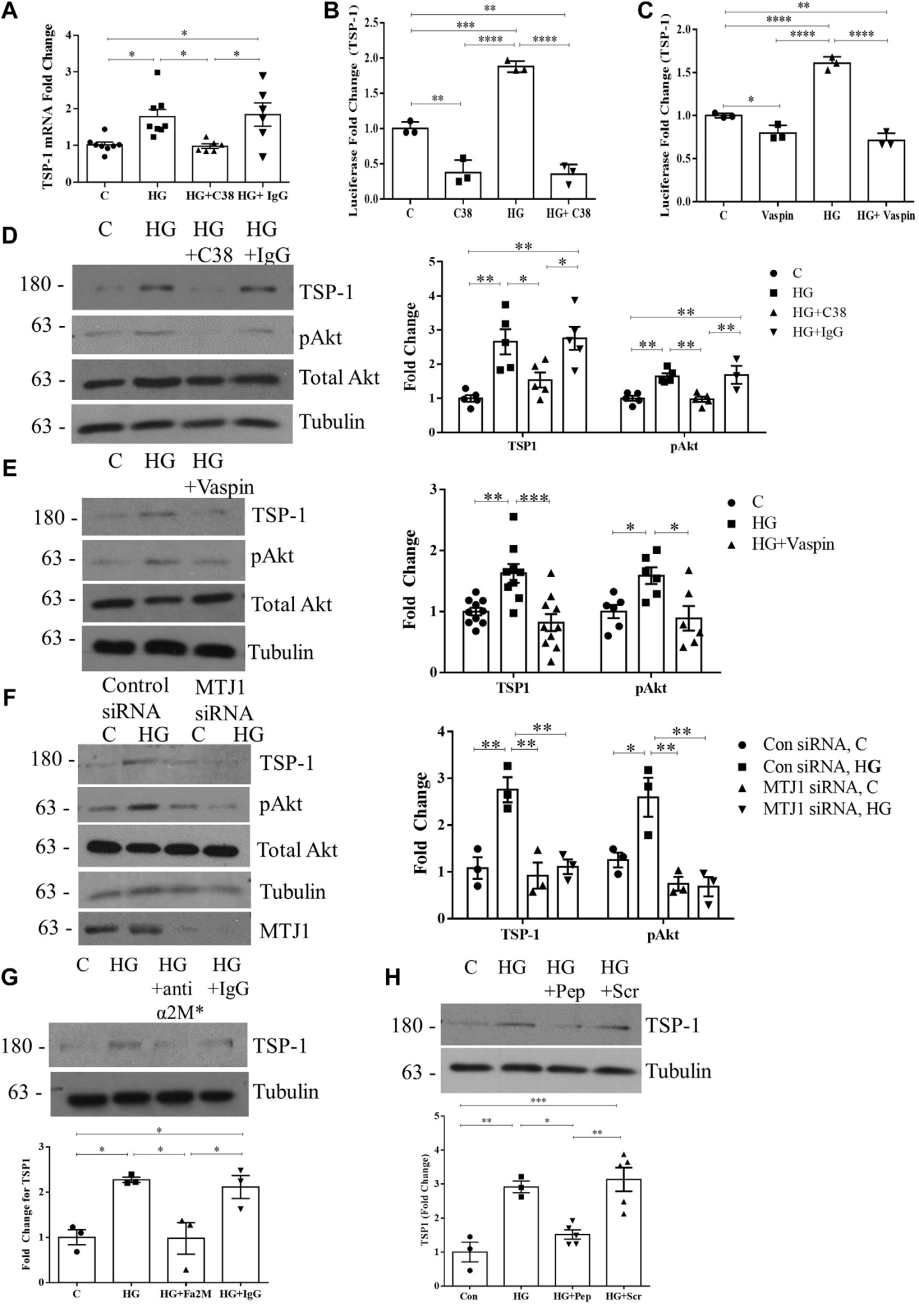
Cell surface GRP78 mediates HG-induced TSP1 expression through Akt activation. **(A)** csGRP78 inhibition using the C38 antibody, but not a control IgG (2 µg for each antibody) prevented HG (24 h)-induced TSP1 transcript upregulation (*n* = 6–8, *p < 0.05). HG (48 h)-induced TSP1 promoter activity, assessed using a luciferase reporter construct, was also inhibited by csGRP78 inhibition using either **(B)** the C38 antibody (*n* = 3, **p < 0.01, ***p < 0.005, ****p < 0.0001) or **(C)** vaspin (*n* = 3, **p < 0.01, ***p < 0.005, ****p < 0.001). Further, csGRP78 inhibition also prevented HG (48 h)-induced Akt activation assessed by its phosphorylation at Ser473 as well as TSP1 upregulation using **(D)** C38, but not control IgG, antibody (*n* = 5, *p < 0.05, **p < 0.01), **(E)** vaspin (*n* = 10, *p < 0.05, **p < 0.01, ***p < 0.005) and **(F)** siRNA knockdown of MTJ1, the chaperone required for cell surface translocation of GRP78 in HG (*n* = 3, *p < 0.05, **p < 0.01). **(G)** Antibody neutralization of the known ligand and activator for csGRP78, α2M*, prevented HG (48 h)-induced TSP1 upregulation. Control IgG had no effect (10 µg for each antibody, *n* = 3, *p < 0.05). **(H)** An inhibitory peptide (Pep) that prevents the interaction between α2M* and csGRP78 also inhibited TSP1 upregulation by HG (48 h), with scrambled peptide (Scr) having no effect (100 nM, *n* = 5, *p < 0.05, **p < 0.01, ***p < 0.005).

We next wished to determine whether csGRP78 was an upstream mediator of TSP1 regulation by HG through its activation of Akt. Cell surface GRP78 signaling was shown to be blocked by the C-terminus targeting GRP78 antibody C38 (Munro and Pelham, 1987; De Ridder et al., 2012) and the adipokine vaspin (visceral adipose tissue-derived serine proteinase inhibitor) in other settings (Nakatsuka et al., 2012; Nakatsuka et al., 2013). We thus used these to assess whether csGRP78 mediates TSP1 upregulation by HG. Figure 3A shows that C38, but not a control IgG, inhibits HG-induced TSP1 transcript upregulation. Similar inhibitory effects were seen on TSP1 promoter activation and increased protein expression by HG with both C38 and vaspin (Figures 3B–E). In Figures 3D, E, we confirmed that both csGRP78 inhibitors prevented HG-induced Akt activation, as assessed by its phosphorylation at Ser473. We further tested the effects of downregulating MTJ1, a co-chaperone required for GRP78 translocation to the cell surface in response to HG (Van Krieken et al., 2019). Short interfering (si)RNA knockdown of MTJ1 inhibited both HG-induced Akt activation and TSP1 upregulation (Figure 3F).

Lastly, we previously showed that the high-affinity ligand for csGRP78, activated alpha 2-macroglobulin (denoted α2M*), mediates PI3K/Akt activation in response to HG through csGRP78 (Trink et al., 2021). Using a neutralizing antibody specific for α2M*, as well as an inhibitory peptide that prevents the interaction between csGRP78 and α2M*, we show that HG-induced TSP1 upregulation is attenuated by α2M* inhibition (Figures 3G, H). Altogether, these data show the importance of α2M*-csGRP78 in the upregulation of TSP1 through Akt signaling in response to HG.

### 3.4 Deposition of TSP1 in the ECM is mediated by csGRP78

**FIGURE 4:**
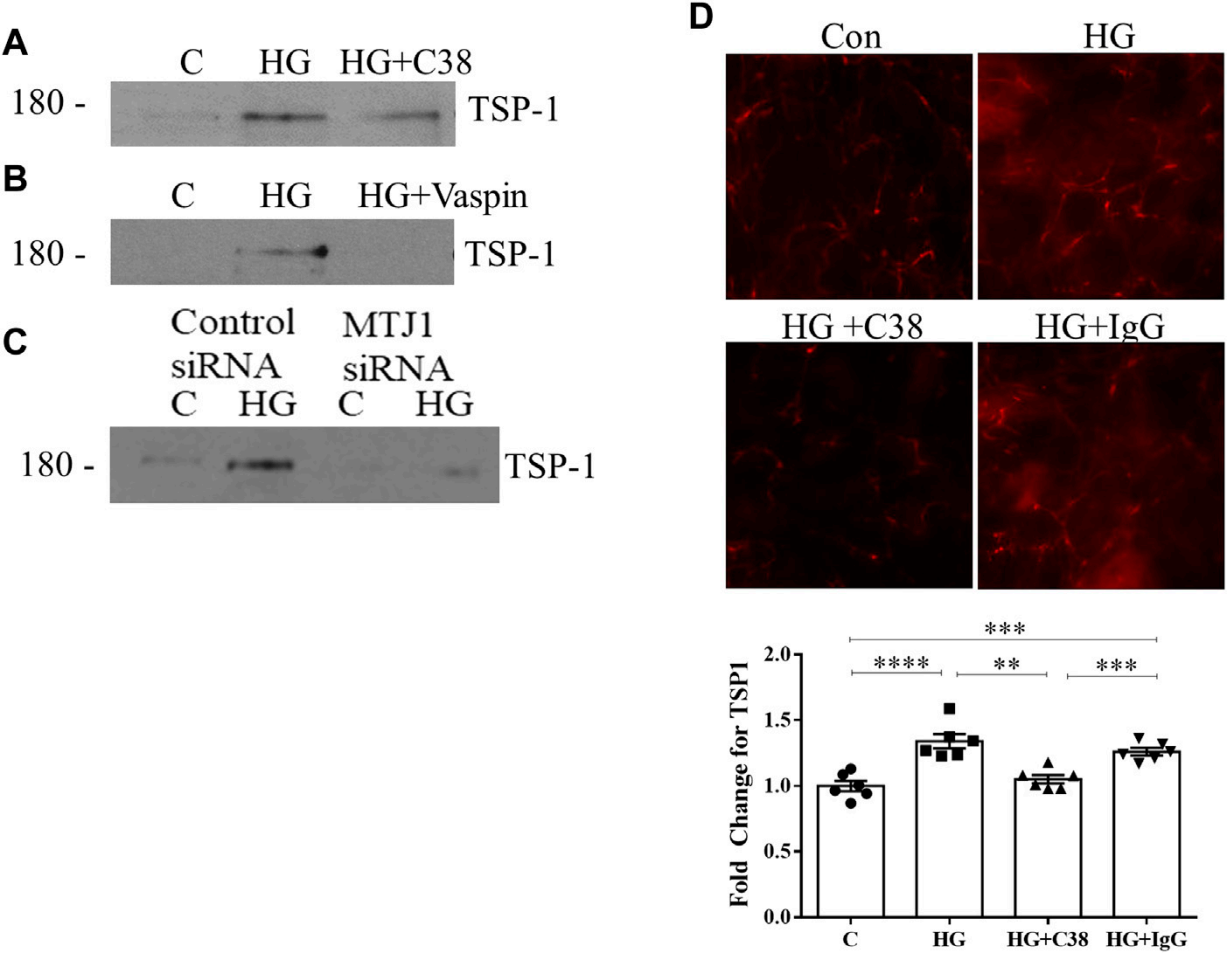
Deposition of TSP1 in the ECM is mediated by csGRP78. ECM was extracted as DOC-insoluble material after HG for 48 h. The increased deposition of TSP1 into the ECM by MC was prevented by csGRP78 inhibition using **(A)** C38 antibody, but not control IgG antibody, **(B)** vaspin and **(C)** MTJ1 knockdown with siRNA (*n* = 3 for each experiment). **(D)** Immunofluorescent staining of remaining ECM. Prior to decellularization, cells were treated with HG for 48 h, causing increased TSP1 deposition into the matrix. This was prevented by C38, but not control IgG (*n* = 6, **p < 0.01, ***p < 0.005, ****p < 0.0001).

Increased deposition of TSP1 into the ECM under HG conditions has previously been shown (Yevdokimova et al., 2001; Xu et al., 2020). Here we investigated whether its deposition is mediated by csGRP78. In Figures 4A, B, inhibition of csGRP78 by C38 or vaspin prevented ECM deposition of TSP1 stimulated by HG. Similar effects were observed with downregulation of MTJ1 to inhibit GRP78 cell surface translocation (Figure 4C). Lastly, the localization of TSP1 in the ECM was visualized using immunofluorescence. As detailed in Methods, cells were removed after treatment, with the remaining DOC-insoluble matrix assessed for TSP1 presence. Inhibition of csGRP78 by the C38 antibody, but not the isotype control IgG, decreased HG-induced TSP1 deposition in the ECM (Figure 4D). Thus, csGRP78 is crucial for TSP1 upregulation as well as ECM deposition in response to HG in MC.

### 3.5 HG-induced TGFβ1 activation requires csGRP78

**FIGURE 5:**
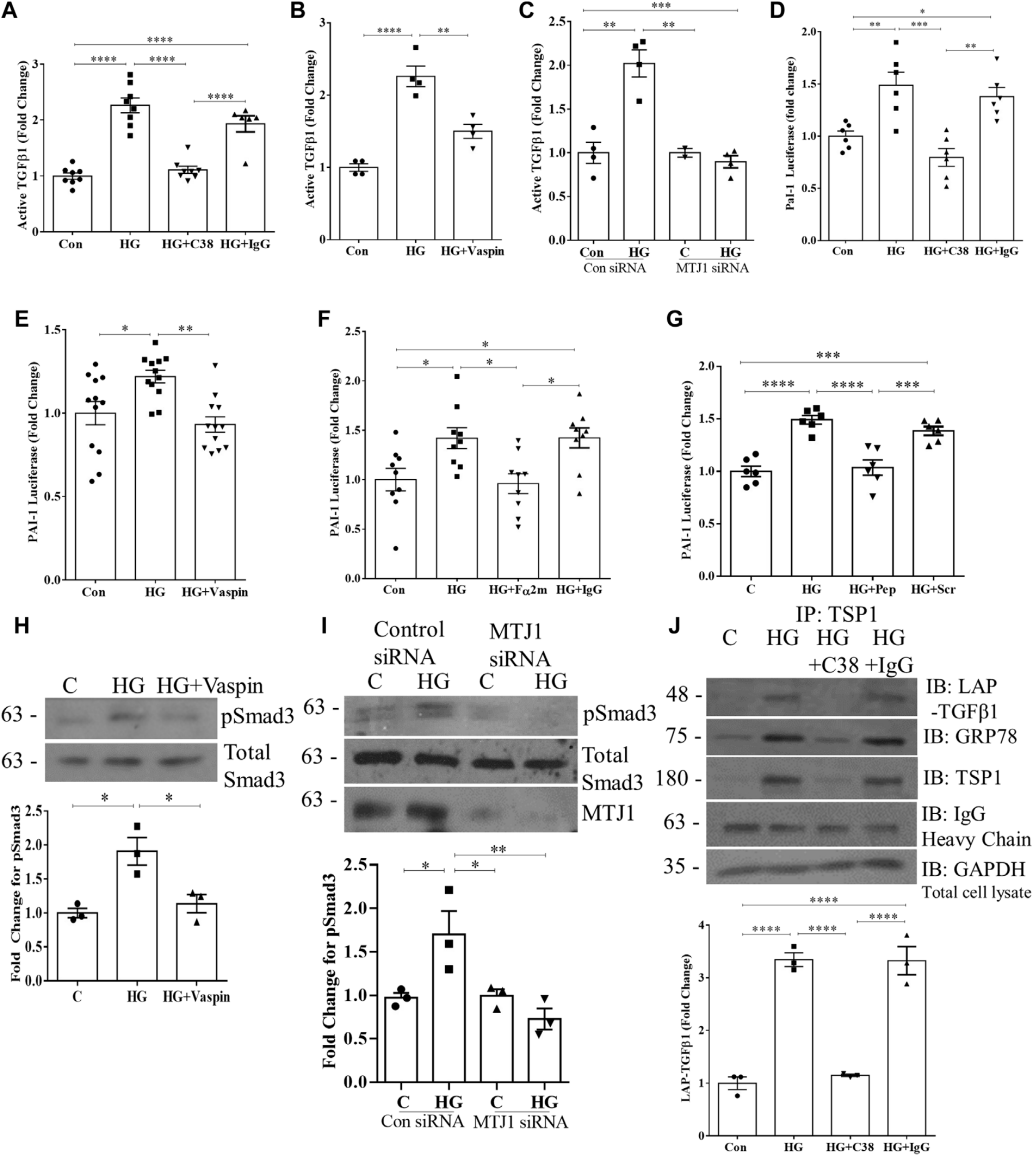
HG-induced TGFβ1 activation requires csGRP78. Assessment of active TGFβ1 in the medium by ELISA showed that csGRP78 inhibition by **(A)** C38, but not a control IgG antibody (*n* = 8, ****p < 0.0001, **(B)** vaspin (*n* = 4, **p < 0.01, ***p < 0.005) or **(C)** MTJ1 siRNA knockdown (*n* = 4, **p < 0.01, ***p < 0.005) prevented HG (48 h)-induced TGFβ1 activation. **(D–F)** MC were co-cultured with mink lung epithelial cells (MLEC) stably transfected with the Smad3-regulated PAI-1 promoter luciferase. The HG (48 h)-induced increase in PAI-1 luciferase activation, reflecting bioactive TGFβ1, was prevented by csGRP78 inhibition with **(D)** C38, but not control IgG antibody or **(E)** vaspin, as well as by α2M* inhibition with **(F)** a neutralizing antibody, but not control IgG (10 µg) or **(G)** a peptide that prevents csGRP78/α2M* interaction (100 nM, *n* = 12, *p < 0.05, **p < 0.01, ***p < 0.005, ****p < 0.0001). In MC, signaling downstream of TGFβ1 was assessed by immunoblotting for Smad3 phosphorylated at Ser473/475. Inhibition of csGRP78 using both **(H)** vaspin and **(I)** MTJ1 siRNA knockdown prevented Smad3 activation in HG (*n* = 3, *p < 0.05, **p < 0.01). **(J)** TSP1 was immunoprecipitated from live cells after HG (48 h) using C38, with IgG used as a control. Immunoblotting shows association with both LAP and csGRP78 in response to HG (*n* = 3, ****p < 0.0001).

Since TSP1 is an important regulator of HG-induced TGFβ1 activation in MC and diabetic kidneys (Poczatek et al., 2000; Wahab et al., 2005; Hohenstein et al., 2008; Lu et al., 2011), and csGRP78 is required for its upregulation, we next investigated whether csGRP78 would be required for TGFβ1 activation by HG. In Figures 5A–C we observed a marked increase in the activation of TGFβ1 in the medium in response to HG. This was prevented by csGRP78 inhibitors C38 antibody and vaspin, as well as the knockdown of MTJ1. We further assessed TGFβ1 activation functionally by using MLECs that are stably transfected with the Smad3-responsive PAI-1 luciferase construct. We first established that PAI-1 luciferase activity is not increased in MLEC in response to HG (not shown). We then co-incubated MLEC with MC. After treatment with HG, increased luciferase activity was observed (Figures 5D–F), reflecting increased bioactive TGFβ1 in the medium. Inhibition of csGRP78 with the C38 antibody (Figure 5D) or vaspin (Figure 5E), as well as inhibition of α2M* using a neutralizing antibody (Figure 5F) or an inhibitory peptide (Figure 5G) all prevented HG-induced TGFβ1 activation. We further confirmed that inhibition of csGRP78 also abrogated TGFβ1 downstream signaling in MC exposed to HG. Figure 5H shows that csGRP78 inhibition with vaspin abrogated HG-induced activation of Smad3 as assessed by its phosphorylation at Ser473/475. Knockdown of MTJ1 similarly prevented Smad3 activation in HG (Figure 5I).

In the ECM, TSP1 dual interaction with LAP and the mature TGFβ1 in its latent complex enables TGFβ1 activation (Crawford et al., 1998; Yevdokimova et al., 2001). We previously showed that HG induces interaction between csGRP78 and LAP (Trink et al., 2022). We thus sought to determine whether csGRP78 could also interact with TSP1. We immunoprecipitated TSP1 from live cell cultures to isolate the cell surface/extracellular TSP1 as outlined in Methods. Figure 5J shows that HG induces an interaction between TSP1, LAP, and csGRP78 which is prevented by csGRP78 inhibition with the C38 antibody. Overall, these data support an important role for csGRP78 in regulating the activation of TGFβ1 in response to HG and suggest that physical interaction at the cell surface is likely important for this regulation.

### 3.6 Overexpression of csGRP78 augments TSP1 production and TGFβ1 activation in HG

**FIGURE 6:**
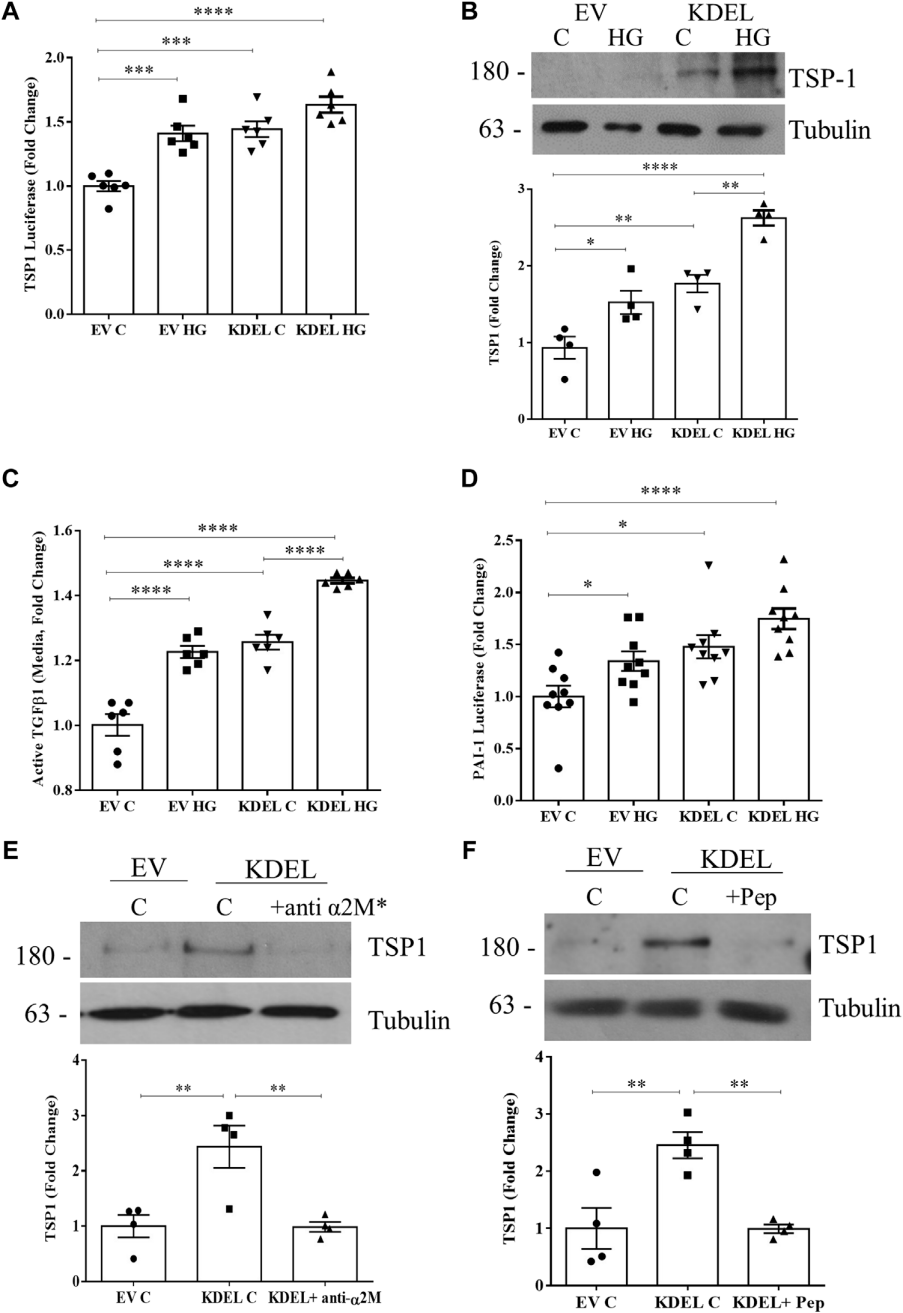
csGRP78 overexpression augments TSP1 production and TGFβ1 activation. Overexpression of GRP78 ΔKDEL, which increases csGRP78, increased basal and HG (48 h)-induced TSP1 **(A)** promoter activity and **(B)** protein expression (*n* = 6, *p < 0.05, **p < 0.01, ***p < 0.005, ****p < 0.0001). **(C)** Activation of TGFβ1 measured by ELISA on medium, as well as its downstream signaling assessed using **(D)** MC co-cultured with MLEC stably expressing PAI-1 luciferase, showed that GRP78 ΔKDEL transfection increased basal and HG (48 h)-induced TGFβ1 activity (*n* = 9, *p < 0.05, ****p < 0.0001). The induction of TSP1 expression by GRP78 ΔKDEL was attenuated by α2M* inhibition using either **(E)** a neutralizing antibody (10 µg) or **(F)** a peptide preventing csGRP78/α2M* interaction (100 nM) (*n* = 4, **p < 0.01).

We have previously shown that overexpressing GRP78 lacking the ER retention sequence KDEL (GRP78 ΔKDEL) increases GRP78 at the cell surface, profibrotic signaling, and matrix production and augments HG responses (Trink et al., 2021). Here we wanted to assess whether csGRP78 overexpression could also augment TSP1 production and TGFβ1 activation. In Figure 6A, we observed increased basal activity of the TSP1 promoter luciferase in cells expressing GRP78 ΔKDEL, at a level similar to that of HG-induced TSP1 luciferase activity. HG further augmented TSP1 promoter activity, although this did not reach statistical significance. We next assessed the effects on the TSP1 protein. Similarly to TSP1 luciferase, protein was increased by GRP78 ΔKDEL alone and further increased with HG (Figure 6B). In parallel, we observed an increase in active TGFβ1 in the medium (Figure 6C) and an increase in TGFβ1 biologic activity assessed using the MLEC system described above (Figure 6D) with GRP78 ΔKDEL alone, also augmented by HG.

Our previous data show that α2M* is required not only for HG responses, but also for csGRP78 effects seen with overexpression of GRP78 ΔKDEL (Trink et al., 2021). To determine whether this extends to TSP1 effects, we treated cells in which csGRP78 was overexpressed using GRP78 ΔKDEL with α2M* inhibitors. Both its neutralizing antibody (Figure 6E) and the inhibitory peptide (Figure 6F) blocked GRP78 ΔKDEL-induced expression of TSP1, showing the importance of this ligand-receptor pair in regulating TSP1 production.

## 4 Discussion

**FIGURE 7:**
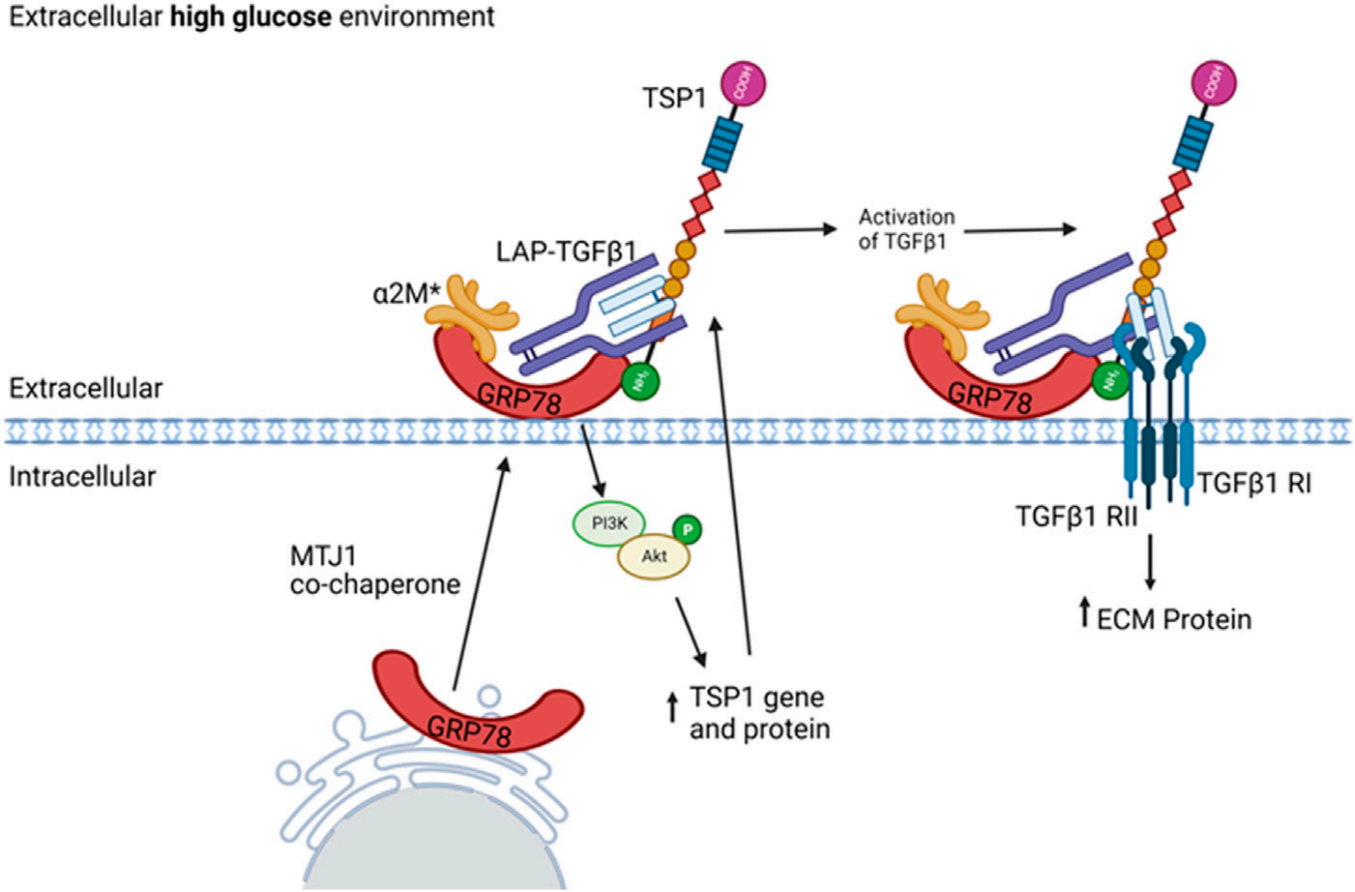
Proposed role for csGRP78 in mediated TGFβ1 activation through regulation of TSP1 in MC. HG promotes GRP78 localization on the cell surface. Increased activation of the protease inhibitor α2M (denoted α2M*) enables its high-affinity interaction with csGRP78. This induces downstream PI3K/Akt activation and consequent TSP1 gene upregulation. The increased TSP1 localizes to the ECM, where others have shown it to interact with latent TGFβ1 and serve as an important regulator of TGFβ1 activation. The nature and role of csGRP78 interaction with this complex requires further study. These data support a role for csGRP78/α2M* in mediating TGFβ1 profibrotic activation in MC by HG, thus highlighting a potential therapeutic target for indirect TGFβ1 inhibition. Created with BioRender.

We have previously shown the *de novo* expression of csGRP78 in DKD and the importance of HG-induced GRP78 translocation to the cell surface to mediate profibrotic signaling in MC (Van Krieken et al., 2019; Trink et al., 2021). While these studies showed that csGRP78 was important for HG-induced TGFβ1 upregulation (Trink et al., 2021; Trink et al., 2022), whether it also regulated activation of TGFβ1 from its latent state was not clear. In these follow-up studies, we chose to focus on TSP1 given its central role in the non-proteolytic activation of TGFβ1 by HG and in DKD, as well as the limited information on how TSP1 production is regulated in this setting. We now present evidence, summarized in Figure 7, that csGRP78 facilitates the production and consequent extracellular deposition of TSP1 through PI3K/Akt signaling in response to HG, with inhibition of this pathway preventing the bioactivation of TGFβ1. These novel data provid further support for the potential therapeutic value of inhibiting csGRP78 to prevent the fibrotic phenotype seen in DKD. Indeed, direct TGFβ1 inhibition is not feasible due to its pleiotropic homeostatic effects. This was shown in a clinical trial evaluating the efficacy of a neutralizing TGFβ1 antibody in patients with DKD, in which dosing that limited adverse effects was ineffective in slowing DKD (Voelker et al., 2017). Indirect methods of TGFβ1 attenuation targeting disease-specific abnormally heightened signaling, while leaving homeostatic signaling undisturbed, are thus of high therapeutic interest. Our study supports csGRP78 as a potential anti-fibrotic therapeutic target that could provide a disease-specific mechanism by which to inhibit TGFβ1 profibrotic signaling in DKD. Current studies are underway to test the efficacy of csGRP78 inhibition in an *in vivo* DKD model.

Previous studies have shown that TSP1 is increased in MC and some non-kidney cells in response to HG as well as other stimuli relevant to DKD such as angiotensin II (Poczatek et al., 2000; Naito et al., 2004; Zhou et al., 2006). Importantly, TSP1 upregulation was also seen in both murine models of DKD and human type 1 and 2 diabetic kidneys (Yevdokimova et al., 2001; Wahab et al., 2005; Hohenstein et al., 2008; Lu et al., 2011; Murphy-Ullrich, 2019). Several studies have shown the pathologic importance of TSP1 as an activator of latent TGFβ1, contributing to the pathogenesis of fibrosis that is characteristic of DKD. Notably, TSP1 was shown to be expressed in a mesangial pattern in DKD (Daniel et al., 2007). In cultured MC, HG-induced TGFβ1 activation was blocked by a peptide that inhibited interaction between TSP1 and latent TGFβ1 (Yevdokimova et al., 2001; Lu et al., 2011). TSP1 knockout mice with type 1 diabetes induced by streptozotocin showed reduced DKD compared to their wild-type counterparts. Less glomerular matrix accumulation, inflammation, and proteinuria were associated with reduced active glomerular TGFβ1 and downstream signaling, with no difference in total TGFβ1 levels (Daniel et al., 2007). In another study, inhibition of latent TGFβ1-TSP1 interaction with a specific peptide attenuated DKD in type 1 diabetic Akita mice, with a reduction in proteinuria, urinary active TGFβ1 and Smad2/3 activation. Importantly, peptide treatment also reduced tubulointerstitial fibrosis, a pathologic finding seen in the later stages of DKD (Lu et al., 2011). TSP1 thus contributes to both early and later-stage DKD development through its regulation of TGFβ1 activation.

Several studies suggest that the important role of TSP1 in kidney fibrosis extends beyond DKD. For example, increased TSP1 was found in the kidneys of remnant rats, a model of reduced kidney mass and fibrosis as is seen in chronic kidney disease of any etiology. This increase preceded and was predictive of the development of tubular interstitial fibrosis (Hugo et al., 2002). Additionally, TSP1 deficiency was found to be protective against the development of proteinuria, fibrosis, and inflammation induced by the chemotherapeutic agent adriamycin in mice (Maimaitiyiming et al., 2016). Interstitial fibrosis that developed following unilateral ureteral obstruction was significantly attenuated by a peptide inhibiting TSP1/latent TGFβ1 interaction, and this was associated with a reduction in active TGFβ1 and Smad activation (Xie et al., 2010). Finally, TSP1 was also found to contribute to the longer-term development of fibrosis after acute injury in an ischemia/reperfusion model, again associated with TGFβ1 activation (Julovi et al., 2020). While requiring assessment, it is probable that csGRP78 is increased in response to various pathogenic stimuli in kidney cells in addition to HG. Indeed, our unpublished data show that angiotensin II, a common pathogenic mediator of fibrosis in diabetic and non-diabetic chronic kidney disease, also induced GRP78 localization to the cell surface in MC. The effect of additional stimuli in MC and other kidney cell types, as well as the localization of GRP78 to the cell surface in various non-DKD models, will be determined in future studies.

While the upregulation of TSP1 by HG in MC and DKD is well documented, the mechanism by which this occurs is less well-defined. An important role for cGMP-dependent protein kinase G (PKG) has been identified, with PKG normally repressing TSP1 transcription. PKG is activated by nitric oxide signaling to activate soluble guanylate cyclase, thereby increasing cGMP levels. In MC exposed to HG, a reduction in nitric oxide in the medium and intracellular cGMP was seen, and a nitric oxide donor or overexpression of a constitutively active PKG prevented HG-induced TSP1 upregulation and TGFβ1 activation (Wang et al., 2002; Wang et al., 2003). TSP1 upregulation in HG was mediated by the transcription factor upstream stimulatory factor 2 (USF2), which under normal glucose conditions is repressed by PKG (Wang et al., 2004). In our studies, we showed an important role for PI3K/Akt activation in TSP1 upregulation. This pathway was also shown to mediate complement-induced TSP1 upregulation and consequent TGFβ1 activation in MC (Wang et al., 2006). Interestingly, increased USF2 production by HG in MC was shown to be dependent on the transcription factor CREB, well known to be activated by Akt phosphorylation (Du and Montminy, 1998; Shi et al., 2008). Future studies will explore whether csGRP78/Akt regulation of TSP1 intersects with PKG/USF2 signaling.

Our previous studies have shown that the high-affinity ligand for csGRP78, α2M*, is locally produced by MC in HG and in DKD to enable PI3K/Akt signaling and downstream ECM production (Trink et al., 2021). We showed not only its activation in the diabetic kidney, but also its upregulation at the protein and transcript levels. Additional data available in the RNA-seq database NephroSeq also show a disease-specific increase in α2M in DKD patients in both glomeruli and the tubulointerstitium compared to healthy control patients (Supplementary Figure S1) (Ju et al., 2013; Ju et al., 2015). Further, α2M was shown to be increased in human endothelial cells and MC in patients with focal segmental glomerular sclerosis (FSGS), another fibrotic disease of the kidney. This study showed that α2M was specifically upregulated in disease and its higher expression was associated with poorer kidney outcomes (Menon et al., 2020), suggesting the importance of α2M* signaling may also be relevant to non-diabetic kidney disease. Our current data extend these observations by showing that α2M* is also required for csGRP78-mediated TSP1 upregulation and TGFβ1 activation in HG. Furthermore, as csGRP78 is positioned entirely extracellularly, we identified integrin β1 as a transmembrane signaling partner for csGRP78 that enables intracellular signal transmission and profibrotic responses (Van Krieken et al., 2019; Trink et al., 2022). Interestingly, TSP1 was also shown to bind integrin β1 (Calzada et al., 2004). Whether activation of TGFβ1 by TSP1 in HG requires binding to integrin β1 and/or csGRP78 needs further study, as do the details of their interaction at a molecular level.

In conclusion, we have shown that HG-induced csGRP78 and α2M* mediate TGFβ1 activation and downstream signaling through the regulation of TSP1. This pathway provides an indirect method of TGFβ1 inhibition that is entirely extracellular, and thus an attractive potential therapeutic target. Further studies will investigate whether inhibition of csGRP78, α2M*, or their interaction is an effective approach to DKD treatment and prevention.

## Data Availability

The raw data supporting the conclusion of this article will be made available by the authors, without undue reservation.
