# Supplementary material for: Cell surface GRP78 regulates TGFβ1-mediated profibrotic responses via TSP1 in diabetic kidney disease
Source: Front Pharmacol. 2023 Feb 24;14:1098321. doi: 10.3389/fphar.2023.1098321 (PMC9998550; doi:10.3389/fphar.2023.1098321)
Supplement: Supplementary file 1 [file Image1.pdf]

A

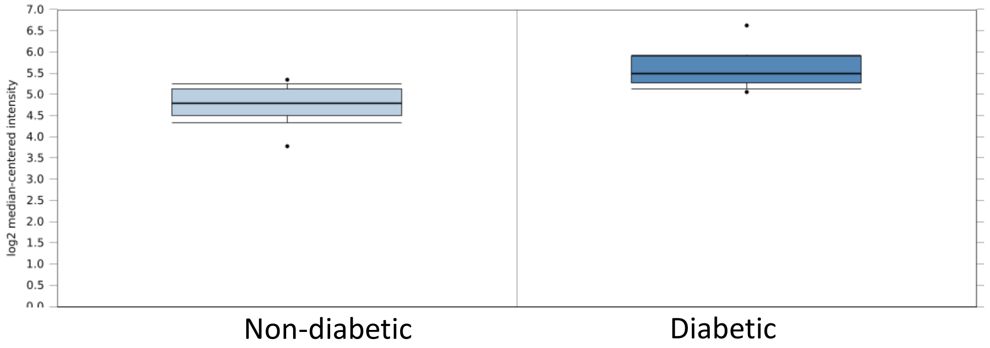

B

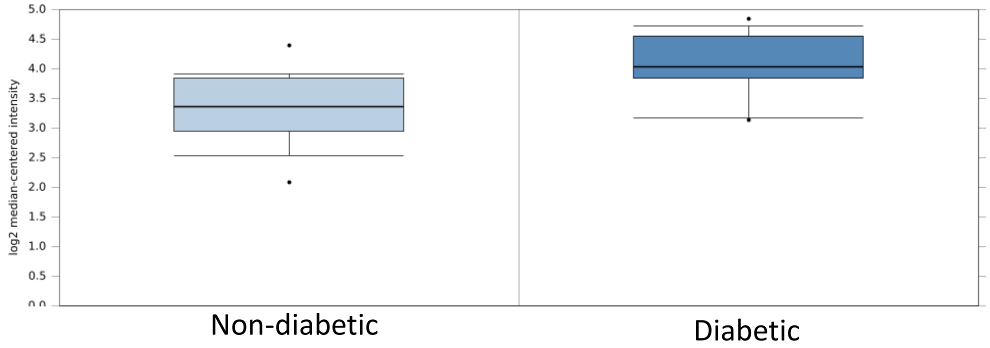

Supplementary Figure 1: NephroSeq data shows increased α2M expression in glomeruli and the tubulointerstitium in DKD. (A) α2M RNA expression was significantly increased in glomeruli from human DKD patients compared to healthy living donors (n=21 for control patients and 12 for DKD patients, ****p< 0.0001). (B) Increased α2M transcript expression was observed in the tubulointerstitum of DKD patients compared to healthy controls (n=31 for control patients and 17 for DKD patients, ****p< 0.0001).
